# Mechanical ventilation enhances extrapulmonary sepsis-induced lung injury: role of WISP1–αvβ5 integrin pathway in TLR4-mediated inflammation and injury

**DOI:** 10.1186/s13054-018-2237-0

**Published:** 2018-11-16

**Authors:** Xibing Ding, Yao Tong, Shuqing Jin, Zhixia Chen, Tunliang Li, Timothy R. Billiar, Bruce R. Pitt, Quan Li, Li-Ming Zhang

**Affiliations:** 1 Department of Anesthesiology, East Hospital, Tongji University School of Medicine, 150 Jimo Road, Pudong, Shanghai, China; 2 Department of Anesthesiology, Xiangya 3rd Hospital, Central South University, Hunan, China; 3 Department of Surgery, University of Pittsburgh School of Medicine, Pittsburgh, PA USA; 4 Department of Environmental and Occupational Health, University of Pittsburgh Graduate School Public Health, Pittsburgh, PA USA; 5 Department of Anesthesiology, University of Pittsburgh School of Medicine, 200 Lothrop St. UPMC MUH N467, Pittsburgh, 15213 PA USA; 6 Department of Anesthesiology, Renji Hospital, Shanghai Jiaotong University School of Medicine, Shanghai, China; 7 Department of Anesthesiology, Cancer Hospital Chinese Academy of Medical Sciences, Shenzhen, China

**Keywords:** Acute lung injury, Integrin, Lipopolysaccharide, Mechanical ventilation, Peritoneal macrophages, Sepsis, Toll-like receptor 4, WISP1

## Abstract

**Background:**

High tidal volume ventilation of healthy lungs or exacerbation of existing acute lung injury (ALI) by more moderate mechanical ventilation (MTV) produces ventilator-induced lung injury. It is less clear whether extrapulmonary sepsis sensitizes the lung to MTV.

**Methods:**

We used a two-hit model of cecal ligation and puncture (CLP) followed 12 h later by MTV (10 ml/kg; 6 h) to determine whether otherwise noninjurious MTV enhances CLP-induced ALI by contrasting wildtype and TLR4^−/−^ mice with respect to: alveolar-capillary permeability, histopathology and intrapulmonary levels of WNT-inducible secreted protein 1 (WISP1) and integrin β5; plasma levels of cytokines and chemokines (TNF-α, IL-6, MIP-2, MCP-1) and intrapulmonary neutrophil infiltration; and other inflammatory signaling via intrapulmonary activation of JNK, p38 and ERK. A separate cohort of mice was pretreated with intratracheal neutralizing antibodies to WISP1, integrin β5 or IgG as control and the presented phenotyping repeated in a two-hit model; there were 10 mice per group in these first three experiments. Also, isolated peritoneal macrophages (PM) from wildtype and TLR4^−/−^, MyD88^−/−^ and TRIF^−/−^ mice were used to identify a WISP1–TLR4–integrin β5 pathway; and the requisite role of integrin β5 in WISP1-induced cytokine and chemokine production in LPS-primed PM was examined by siRNA treatment.

**Results:**

MTV, that in itself did not cause ALI, exacerbated increases in alveolar-capillary permeability, histopathologic scoring and indices of pulmonary inflammation in mice that previously underwent CLP; the effects of this two-hit model were abrogated in TLR4^−/−^ mice. Attendant with these findings was a significant increase in intrapulmonary WISP1 and integrin β5 in the two-hit model. Anti-WISP1 or anti-integrin β5 antibodies partially inhibited the two-hit phenotype. In PM, activation of TLR4 led to an increase in integrin β5 expression that was MyD88 and NF-κB dependent. Recombinant WISP1 increased LPS-induced cytokine release in PM that was inhibited by silencing either TLR4 or integrin β5.

**Conclusions:**

These data show for the first time that otherwise noninjurious mechanical ventilation can exacerbate ALI due to extrapulmonary sepsis underscoring a potential interactive contribution of common events (sepsis and mechanical ventilation) in critical care, and that a WISP1–TLR4–integrin β5 pathway contributes to this phenomenon.

**Electronic supplementary material:**

The online version of this article (10.1186/s13054-018-2237-0) contains supplementary material, which is available to authorized users.

## Introduction

Mechanical ventilation (MV) is well known to cause an iatrogenic syndrome of ventilator-induced lung injury (VILI). The pathophysiology of VILI includes intrapulmonary inflammatory cell infiltrates, increased vascular permeability and pulmonary edema, and it may occur in ventilation of a healthy lung or worsening of preexisting and coexisting injury [1]. Sensitization of VILI secondary to preexisting acute lung injury (ALI) due to pneumonia [2, 3], intratracheal endotoxin [4–7] or sterile injury [8–10] has provided preclinical evidence of a two-hit model.

Although extrapulmonary endotoxemia combined with noninjurious mechanical ventilation leads to VILI [11, 12], evidence of such a two-hit phenomenon in experimental extrapulmonary bacterial sepsis is less clear. Ventilating rodents after polymicrobial sepsis due to cecal ligation and puncture (CLP) [13–15] has produced equivocal results regarding sensitization to VILI. Mechanical ventilation with injurious high VT (30–40 ml/kg) exacerbated 48 h of CLP-induced lung injury in rats [16]; shorter periods of CLP were not associated with subsequent exacerbation of VILI in mice [17] or rats [9, 18] although such overall injury was accelerated in the latter. Lower VT (15–20 ml/kg) did not exacerbate CLP-induced ALI in intact rats [9] or isolated perfused rat lungs [19]. Since both CLP and VILI have a common TLR4-mediated pathway to inflammation and injury [5, 20–22] it seems plausible that mechanical ventilation could exacerbate CLP-mediated events within the lung and thus differences are likely to be secondary to variables in experimental protocols (degree of preexisting lung injury, volume and duration of ventilation) as suggested by Yehya et al. [18].

In the current study, we examined the effect of prolonged (6 h), otherwise noninjurious [4] moderate VT ventilation (MTV) in mice with preexisting mild ALI after CLP (12 h). We focused on a novel WNT1 inducible secreted protein (WISP1 or CCN4; also referred to as WNT1 inducible signaling protein-1)–integrin β5 pathway of TLR4-mediated pulmonary inflammation and injury in this two-hit model as we previously noted key roles for WISP1 and beta integrins in VILI [21] and CLP [22, 23] mediated ALI. The mechanism by which WISP1 acts in CLP and/or VILI remains unclear. WISP1 appears, however, to be a modulator of TLR4–CD14 signaling and is known to signal through integrins [24] although the precise integrin is unclear. Although several integrins are important in ALI [25], integrin β5 is a central regulator of increased permeability in VILI [26] and CLP [27].

Thus, the overall hypotheses of this study were that: prolonged ventilation with otherwise noninjurious moderate VT exacerbates ALI in extrapulmonary sepsis; WISP1 and integrin β5 contribute to this two-hit model (i.e., CLP + MTV); and TLR4 is central to the WISP1–TLR4–integrin β5 proinflammatory pathway.

## Methods

### Experimental protocols

Animal protocols were approved by the Animal Care and Use Committee and experiments were performed in strict adherence to NIH Guidelines and followed current guidelines for preclinical models in research. Details of materials and methods are provided in Additional file 8: Materials and Methods) and experimental protocols are outlined for intact mice (Additional file 1: Figure S1) and cultured PM (Additional file 2: Figure S2) including: MTV and CLP-induced lung damage via TLR4-dependent, WISP-1 and integrin β5 contributory fashion in two-hit lung damage; MTV and CLP-induced changes in circulating levels of cytokines and chemokines, and an association with increased neutrophil infiltration of the lungs of intact mice; other inflammatory signaling (pJNK, p38, pERK) pathways in lungs of mice after CLP and MV; the mechanism of upregulation of integrin β5 in LPS-treated PM isolated from wildtype, TLR4 null, Myd88^−/−^ and TRIF^−/−^ mice; and the requisite role of integrin β5 in WISP1-induced cytokine and chemokine production in LPS-primed PM.

### In-vivo experimental animal model

C57BL/6 mice (8–10 weeks old, male) were purchased from Jackson Laboratory and TLR4^−/−^ mice were used as described previously [21, 22]. Forty wildtype mice were prospectively randomized to one of four groups (*n* = 10 per group): spontaneous breathing (sham control), spontaneous breathing with CLP; mechanical ventilation; or CLP and MTV. Mild sepsis was induced by CLP [13, 14] as modified by Ding et al. [23, 28] and MTV (10 ml/kg; 150/min, zero positive end-expiratory pressure) was performed in anesthetized mice alone or after CLP (12 h). A cohort (*n* = 20) of TLR^−/−^ mice underwent sham and spontaneous breathing (*n* = 10; control) or CLP and MTV as already described (*n* = 10). In separate cohorts, mice were intratracheally administered anti-WISP1 (*n* = 10), anti-integrin β5 (*n* = 10) or serum IgG (*n* = 10; 0.5 μg/g in 50 μl PBS) after CLP but before MTV. Phenotypic changes due to CLP, MTV or their combination included histopathology (scored by a pathologist blinded to experiments), alveolar-capillary permeability (Evans Blue albumin [21]) and inflammation as assessed by plasma levels of cytokines and chemokines, neutrophil immigration in the lung (flow cytometry) and intrapulmonary MAP kinase activation (phosphorylation status of JNK, ERK and p38).

### In-vitro studies

Isolation and culture of peritoneal macrophages (PM) from wildtype, TLR4^−/−^, MyD88^−/−^ or TRiF^−/−^ mice was performed as described previously [21, 22]. Cells were treated with LPS and/or costimulated with recombinant WISP1 (rWISP1) and cytokine production was assessed in conditioned medium of wildtype cells or after transfection with 50 nM small interfering RNA (siRNA) for integrin β5 (sc-35681; Santa Cruz) or scrambled siRNA. The role of TLR4 signaling was assessed by comparing the response of PM isolated from wildtype vs TLR4^−/−^, Myd88^−/−^ or TRIF^−/−^ mice.

### Histological examination

Lung tissue samples were fixed in 4% paraformaldehyde in PBS overnight at 4 °C and processed as described previously [22] including semiquantitative histopathology (H&E; light microscopy) by a pathologist blinded to the experimental group.

Western blot analysis of WISP-1 and integrin β5 was performed as described previously [21, 22]. Plasma or conditioned medium were assayed for cytokines and chemokines using commercially available ELISA reagents for TNF-α, IL-6, MIP-2 and MCP-1.

### Flow cytometry

The lung was enzymatically digested and mechanically dissociated (MACS dissociator) and single cell suspensions were isolated by passing the suspension through a 70-μm filter. Cells were stained with mAbs specific to Fixable Viability Dye eFluor® 506, CD45, CD11b, Ly6G for 30 min at 4 °C and fixed with 2% paraformaldehyde for 10 min at 4 °C. An LSR II (Becton Dickinson) was used for flow cytometry and data were analyzed with FlowJo software.

### Alveolar-capillary permeability

Evans blue albumin (EBA; 0.5%, 25 mg/kg body weight) was injected into the internal jugular vein 1 h before euthanasia and lung harvesting. Blood samples and lung tissue were obtained and processed as described previously [21, 22] and the EBA permeability index was calculated by dividing pulmonary EBA absorbance at 620 nm/g of lung tissue by plasma EBA absorbance at 620 nm.

### Immunofluorescence staining of cells and florescence microscopy

PM were cultured for a defined time period, fixed in 4% paraformaldehyde in PBS for 15 min. Cells were washed in PBS, permeabilized using 0.1% Triton X-100, blocked with 5% BSA for 45 min and sequentially administered primary antibody and secondary antibody (Alexa-488-conjugated donkey anti rabbit secondary antibody). Nuclei were stained with DAPI (Thermo Fisher Scientific) and cells were examined and recorded using EVOS FL fluorescence microscopy (immunofluoresence analysis; Thermo Fisher Scientific).

Reagents are described in Additional file 8: Materials and Methods.

### Data analysis and statistics

Data are presented as the mean ± SEM of the indicated number of experiments and analyzed using one-way and two-way ANOVA; post-hoc testing was performed using the Bonferroni modification of the *t* test. The individual studies performed throughout this work represent five independent studies. Power analyses were performed by using type I error probability of 0.05, with a power of 0.9, to determine the sample size necessary to reject the null hypothesis. All statistical analyses were carried out using the GraphPad Prism 5 program. *P* < 0.05 was considered statistically significantly.

## Results

**Fig. 1:**
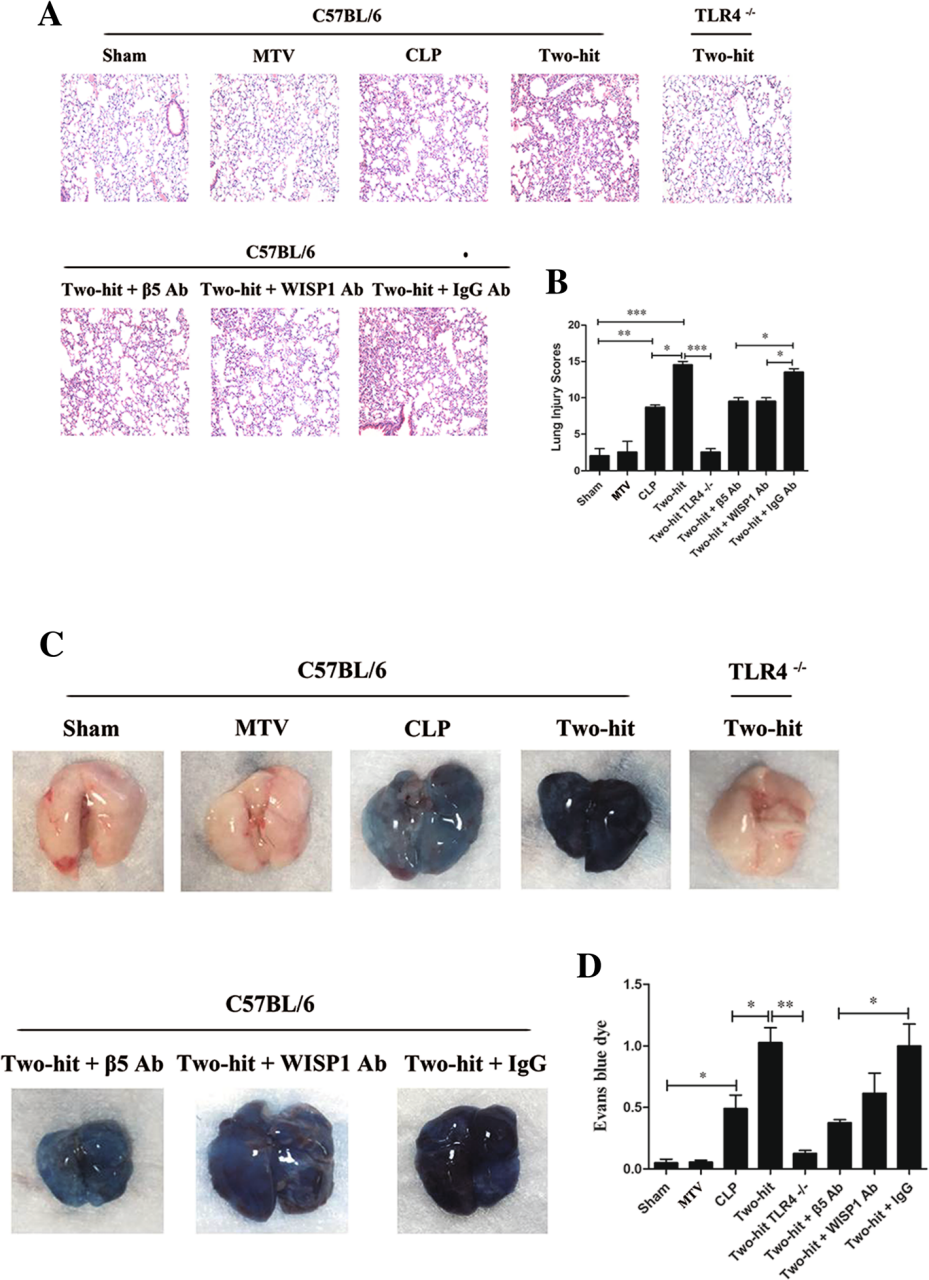
MTV enhances CLP-induced lung damage via TLR4-dependent WISP1–integrin β5 pathway. As shown in Additional file 1: Figure S1, mice lung tissue samples in eight mice groups were fixed and stained with hematoxylin–eosin for histological analysis (**a**) and lung injury score (**b**). Gross lung image in each group (**c**) and vascular permeability evaluated by Evans blue dye (**d**). Mice receiving combination of CLP + MTV (two-hit model) compared to mice subjected to CLP alone for 18 h or sham operation followed by 6 h of MTV. Two-hit model in wildtype mice compared to subgroup of TLR4^−/−^ mice or wildtype mice that received intratracheally neutralizing antibodies to either integrin β5 (β5 Ab) or WISP1 (WISP1 Ab) or a control antibody (IgG Ab) during mechanical ventilation. *P < 0.05; **P < 0.01; ***P < 0.001. CLP cecal ligation and puncture, MTV moderate tidal ventilation, TLR4 toll-like receptor 4, WISP1 WNT1 inducible secreted protein

**Fig. 2:**
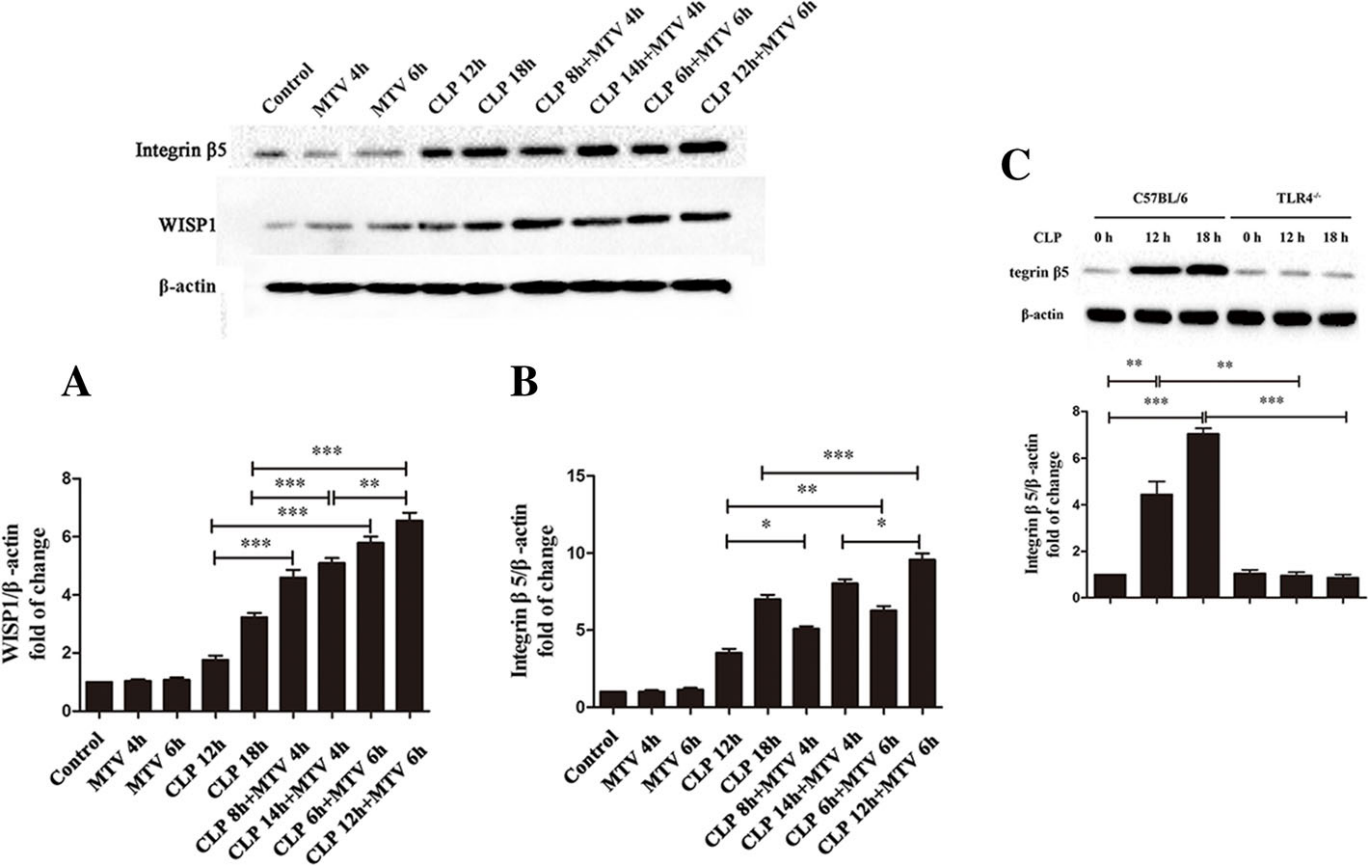
MTV enhances CLP-induced WISP1 and integrin β5 expression via TLR4-dependent pathway. WISP1 protein level (**a**) and integrin β5 expression (**b**) in lungs from each group of mice shown by western blot. MTV did not affect both levels and CLP led to small increases but two-hit model increased very significantly. Integrin β5 expression in lungs induced by CLP demonstrated in time-dependent fashion from wildtype mice (C57BL/6) but not TLR4 ^−/−^ mice (**c**). Corresponding actin identified for normalizing densitometry. *P < 0.05; **P < 0.01; ***P < 0.001. CLP cecal ligation and puncture, MTV moderate tidal ventilation, TLR4 toll-like receptor 4, WISP1 WNT1 inducible secreted protein

CLP alone led to modest lung injury as demonstrated by histology (Fig. 1a, b) and a significant increase in alveolar-capillary permeability (Fig. 1c, d). MTV alone had no impact on lung injury or permeability, but when applied after CLP it markedly enhanced both the lung injury score and alveolar-capillary permeability. The histopathologic and permeability changes in the two-hit model were completely abrogated in TLR4^−/−^ mice and partially (but significantly) reduced in cohorts of mice receiving antibodies to either WISP1 or integrin β5. In wildtype mice, we noted that: MTV did not affect intrapulmonary levels of either WISP1 or integrin β5; CLP led to small but significant increases in either WISP1 or integrin β5; and the two-hit model increased either of these molecules 2–3× more than CLP alone (Fig. 2a, b). We previously noted that high VT ventilation increases in WISP1 were abrogated in TLR4^−/−^ mice [21] and we now note (Fig. 2c) that CLP-induced increases in integrin β5 are abrogated in TLR4^−/−^ mice.

**Fig. 3:**
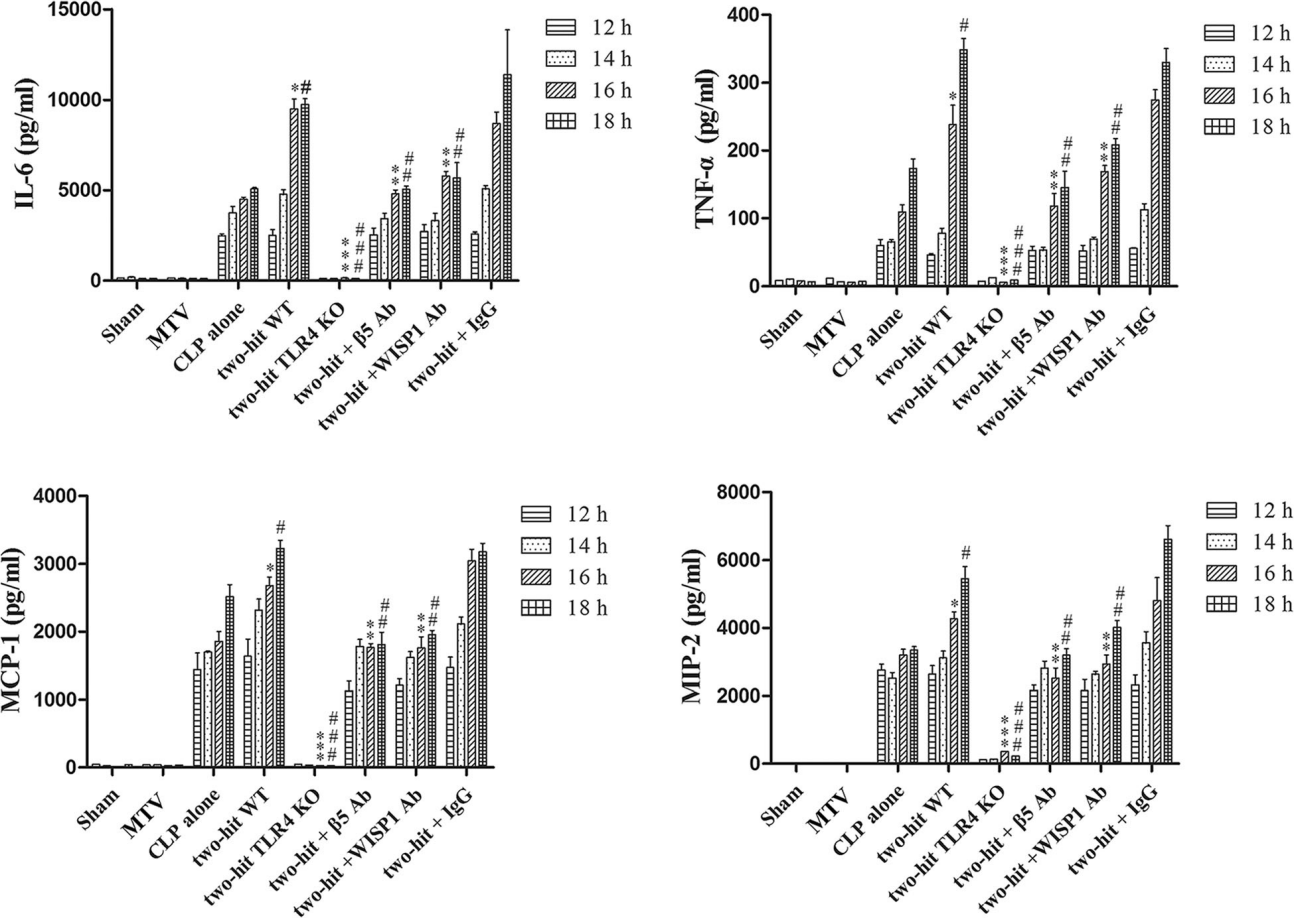
MTV increases circulating levels of cytokines and chemokines. Cytokines (TNF-α and IL-6) and chemokines (MIP-2 and MCP-1) in plasma detected by ELISA. Mice receiving combination of CLP + MTV (two-hit model) compared to mice subjected to CLP alone or sham operation followed by MTV. Two-hit model in wildtype mice compared to subgroup of TLR4^−/−^ mice (TLK4 KO) or wildtype (WT) mice that received intratracheally neutralizing antibodies to either integrin β5 (β5 Ab) or WISP1 (WISP1 Ab) or a control antibody (IgG) followed with CLP 12, 14, 16 and 18 h and MTV 0, 2, 4 and 6 h, respectively. CLP alone induced expected increase in circulating cytokines and chemokines but not induced by MTV alone. Two-hit model (CLP + MTV) increased cytokines and chemokines by MTV in time-dependent manner whereas deletion of TLR4 prevented these increases and inhibition of WISP1 or integrin β5 with neutralizing antibodies also blocked increases induced by MTV. *P < 0.05 compared with CLP alone at 16 h; **P < 0.05 compared with two-hit WT at 16 h; ***P < 0.05 compared with two-hit WT; ^#^*P* < 0.05 compared with CLP alone at 18 h; ^##^*P* < 0.05 compared with two-hit WT at 18 h; ^###^ *P* < 0.05 compared with two-hit WT at 18 h. CLP cecal ligation and puncture, IL interleukin, MCP-1 monocyte chemoattractant protein-1, MIP-2 macrophage inflammatory protein-1, MTV moderate tidal ventilation, TLR4 toll-like receptor 4, TNF-α tumor necrosis factor alpha, WISP1 WNT1 inducible secreted protein

CLP increased circulating cytokines and chemokines whereas MTV alone did not; the combination of MTV and CLP, however, caused levels of all four mediators to progressively rise above levels measured with CLP alone (Fig. 3). Deletion of TLR4 prevented increases in cytokines and chemokines in the two-hit model and inhibition of WISP1 or integrin β5 blocked further increases induced by MTV (Fig. 3).

**Fig. 4:**
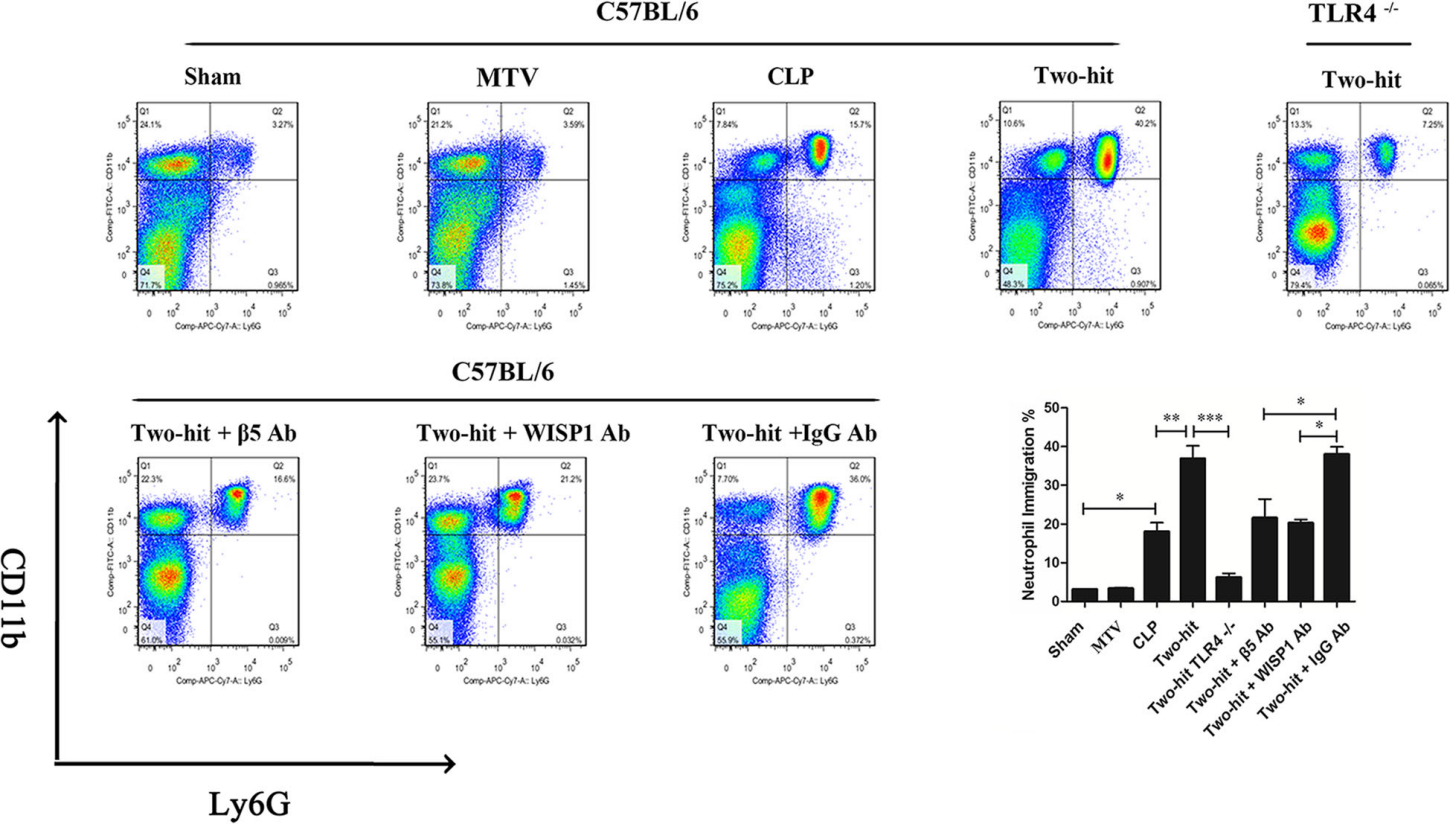
MTV increases PMN infiltration in lungs of septic mice. Neutrophil immigration in lung detected by flow cytometry. Mice receiving combination of CLP + MTV (two-hit model) compared to mice subjected to CLP alone for 18 h or sham operation followed by 6 h of MTV. Two-hit model in wildtype mice compared to subgroup of TLR4 null mice or wildtype mice that received intratracheally neutralizing antibodies to either integrin β5 (β5 Ab) or WISP1 (WISP1 Ab) (or control antibodies (IgG Ab)). Neutrophil influx by quantifying percent of CD11b^+^ and Ly6G^+^ cells in CD45^+^ cell population isolated from lung homogenates. CLP significantly increased percent of PMN while MTV had no impact on percent PMN in lung; CLP and MTV (two-hit model) significantly further increased neutrophil immigration in lung whereas TLR4 deletion completely prevented change in percentage of PMN induced by CLP + MTV compared to this two-hit model in wildtype mice. Blocking either WISP1 or integrin β5 partially prevented increase in percentage of PMN induced in two-hit model compared to combined CLP and MTV in mice receiving control IgG antibody. *P < 0.05; **P < 0.01; ***P < 0.001. CLP cecal ligation and puncture, MTV moderate tidal ventilation, TLR4 toll-like receptor 4, WISP1 WNT1 inducible secreted protein

CLP significantly increased neutrophil influx in the lung while MTV had no impact; CLP and MTV further significantly increased neutrophil immigration (Fig. 4) that was abolished in TLR4 null mice. Blocking WISP1 or integrin β5 partially prevented the increase in the percentage of PMN induced in the two-hit model compared to combined CLP and MTV in mice receiving control IgG antibody.

We sought further evidence that MTV directly enhanced inflammation in the lungs of septic mice by measuring levels of activated JNK, p38 and ERK MAP kinase. Six hours of MTV had no effect on MAP kinase activation but significantly promoted MAP kinase activation in mice previously subjected to CLP (Additional file 3: Figure S3). TLR4 deletion prevented the increases in MAPK activation in CLP-treated and CLP + MTV-treated mice. Blocking WISP1 or integrin β5 also prevented the increase in MAP kinase phosphorylation induced by MTV in CLP mice.

To explore the mechanism of integrin β5 upregulation by TLR4, we exposed peritoneal macrophages (PM) to LPS and found that ultrapure LPS induced a time and concentration-dependent increase in surface integrin β5 expression (Additional file 4: Figure S4). The increase in integrin β5 expression was TLR4 and MyD88 dependent, but TRIF independent (Additional file 5: Figure S5). We also found that integrin β5 upregulation by LPS was NF-κB dependent (Additional file 6: Figure S6).

**Fig. 5:**
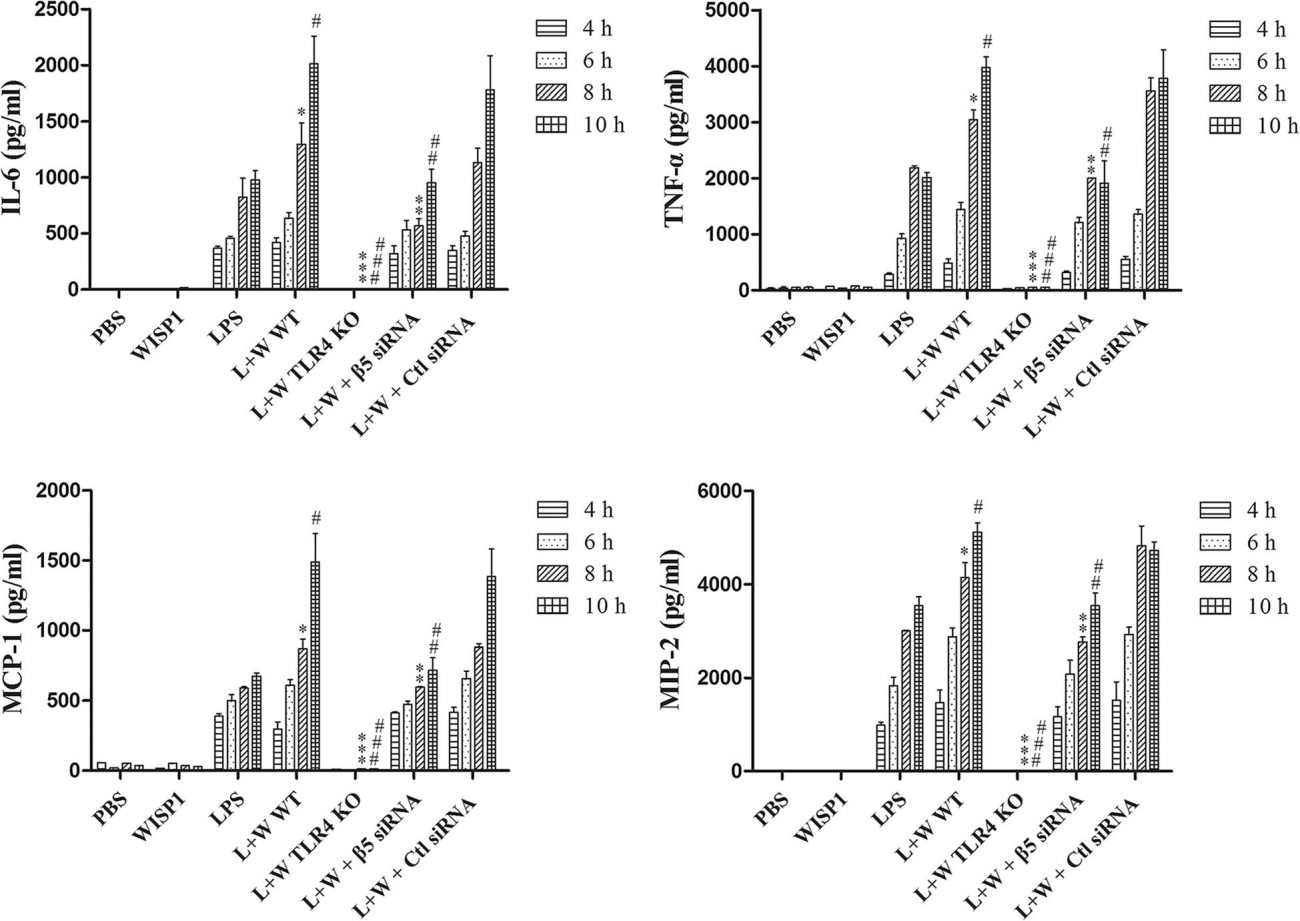
WISP1-induced cytokine and chemokine production in LPS-primed peritoneal macrophages requires integrin β5. Peritoneal macrophages (PM) treated with LPS (0.1 μg/ml) followed by WISP1 (10 μg/ml) exposure at 4 h. Supernatants collected at 2 h intervals from 4 to 10 h following WISP1 stimulation. Cytokines (TNF-α and IL-6) and chemokines (MIP-2 and MCP-1) in supernatant detected by ELISA. PM receiving combination of LPS + WISP1 (L + W) from wildtype mice (WT) compared to PM from subgroup of either TLR4 ^−/−^ mice or wildtype mice receiving integrin β5 siRNA transfection (or control siRNA) subjected to LPS or WISP1. LPS-induced increases in IL-6, TNF-α, MIP-2 and MCP-1 evident within 4–10 h and addition of WISP1 induced further increases in all four mediators. Suppression of integrin β5 expression prevented WISP1-induced enhancement of LPS induction in four mediators. *P < 0.05 compared with LPS alone at 8 h; **P < 0.05 compared with L + W WT at 8 h; ***P < 0.05 compared with L + W WT; ^#^*P* < 0.05 compared with LPS alone at 10 h; ^##^*P* < 0.05 compared with L + W WT at 10 h; ^###^ *P* < 0.05 compared with L + W WT at 10 h. Ctl, control, IL interleukin, KO knockout, LPS lipopolysaccharide, MCP-1 monocyte chemoattractant protein-1, MIP-2 macrophage inflammatory protein-1, PBS phosphate buffered saline, siRNA small interfering RNA, TNF-α tumor necrosis factor alpha, WISP1 WNT1 inducible secreted protein

LPS-induced increases in IL-6, TNF-α, MIP-2 and MCP-1 in medium of isolated PM were evident within 4–10 h and the addition of rWISP1 induced further increases in all four mediators (Fig. 5). No increase in any of the mediators could be seen in PM from TLR4^−/−^ mice. Suppression of integrin β5 expression with siRNA (Additional file 7: Figure S7) prevented the WISP1-induced enhancement of LPS-induced IL-6, TNF-α, MIP-2 and MCP-1 released by PM (Fig. 5).

## Discussion

In the current study, MTV did not cause ALI, but exacerbated CLP-mediated increases in alveolar-capillary permeability and indices of pulmonary inflammation (histopathology, cytokines, chemokines, neutrophil influx and activation of MAPK) in wildtype mice: the effects of this two-hit model were completely abrogated in TLR4 null mutants and partially inhibited by neutralizing antibodies to either WISP1 or integrin β5. In PM, activation of TLR4 led to an increase in integrin β5 expression and rWISP1 increased LPS-induced cytokine release in PM that could be inhibited by silencing either TLR4 or integrin β5. Collectively, these data show: that prolonged MTV ventilation exacerbates ALI caused by extrapulmonary sepsis; and an important positive feedback role for the WISP1–integrin β5 pathway in TLR4-mediated exacerbations to this two-hit model.

Mechanical ventilation with high VT is well known to injure healthy lungs and a consensus from experimental and clinical conditions supports the hypothesis that mechanical ventilation can worsen injury in previously damaged lungs [1, 11, 29–31]. Although systemic [11] endotoxin interacts with mechanical ventilation in producing ALI [16], there is a lack of consensus from studies directed at identifying extrapulmonary bacterial sepsis as a sensitizing condition to VILI. CLP remains the gold standard of experimental polymicrobial sepsis [15] but ventilating rodents after CLP has produced equivocal results regarding sensitization to VILI [9, 18, 19]. Nin et al. [16] reported that mechanical ventilation aggravated CLP-induced multiorgan dysfunction in rats but the investigators used prolonged CLP (24–48 h) and high VT (35 ml/kg). Others have used lower VT ventilation after CLP and did not observe worsening of lung injury [9]. Uematsu et al. [17] noted that high VT ventilation (40 ml/kg) after CLP increased mediator release but did not affect pulmonary function. Yehya et al. [18] showed that high VT (30 ml/kg) accelerated lung injury secondary to previous CLP in rats but the endpoints of injury (lung compliance, pulmonary edema, oxygenation and computed tomography of micro-CT scans) reached the same pathophysiology as from CLP alone. These authors highlighted subtleties in the degree of initial injury with CLP and the magnitude and duration of mechanical ventilation, and speculated that prolonged ventilation with lower VT may indeed enhance lung injury after CLP. In this regard, we unequivocally note that otherwise noninjurious MTV for a prolonged (6 h) time exacerbated underlying CLP-induced ALI. Indeed, whether CLP in itself causes ALI in mice is conjectural. Iskander et al. [32] clearly showed that pulmonary injury after CLP in mice cannot be considered the etiology of death in the acute phase. The authors did note that a severe model of sepsis with significant mortality could show signs of lung injury. We reported previously [22, 23, 28] that the current model of CLP was associated with 80% mortality in 72 h and this presumably accounted for the modest but significant ALI noted at the earlier time period in the current study. Furthermore, we noted that 6 h of MTV (10 ml/kg) without PEEP was void of significant lung injury. In this regard, our model of effects of mechanical ventilation reproduces previous experience of lack of injury with MTV (10 ml/kg, zero PEEP) [4, 28], and was somewhat similar to the results from Hegeman et al. [33] who noted little evidence of ALI in mice after 5 h of mechanical ventilation with 7 ml/kg and 3 cmH_2_O of PEEP or 5 h of MV with 15 ml/kg and zero PEEP. These authors did report VILI under both conditions at a longer time period (12 h) [33].

We [21, 34] and others [5, 35] have noted the important role of TLR4 in experimental VILI. We also noted [22, 23, 28] that a considerable component of pathophysiology, inflammation and injury of CLP was due to TLR4 activation (and CD14). The injury, neutrophil sequestration and inflammation due to combined effects of CLP and MTV in the current study were all abrogated in TLR4 null mice.

WISP1 is a secreted matricellular protein involved in cell adhesion, migration, differentiation, proliferation and survival [36]. From an unbiased haplotype association mapping in inbred strains of mice, we identified WISP1 as a candidate gene associated with VILI [21]. We subsequently identified a role for WISP1 in CLP-induced ALI [22, 23]. WISP1 was first noted in the lung to be a component of bleomycin-induced lung injury and fibrosis [37], and subsequently has been reported to be important in epithelial–mesenchymal transition [38], airway remodeling [39] and proliferation of fibroblasts in the context of lung fibrosis [40]. In-vitro mechanical stretch of type II epithelial cells activated innate immunity and increased WISP1 expression, providing fundamental support for its involvement in VILI [41]. We noted that intrapulmonary WISP1 is elevated in VILI [21], CLP [22, 23], combined poly(I:C) and mechanical ventilation [42], and in the current report in combined CLP and MTV; neutralizing antibodies to WISP1 partially reduced lung injury and inflammation in all of these conditions. The potential convergence of WNT/β-catenin signaling and WISP1 adds to its importance in VILI [43] as well as WNT-mediated lung epithelial cell repair [44].

The mechanism by which WISP1 acts in CLP and/or VILI remains unclear. It appears, however, to be a modulator of TLR4–CD14 signaling and is known to signal through integrins [24]. WISP1 coimmunoprecipitated with active, glycosylated TLR4 in lungs of mice subjected to high VT ventilation and rWISP1 augmented LPS-induced TNF-α release in a TLR4–CD14-dependent fashion in PM [21]. The RGD peptide-sensitive response of intact mouse lungs to CLP and the coimmunoprecipitation of WISP1–integrin β6 in lungs of these mice [23] suggested the presence of the WISP1–integrin β6 pathway in mediating TLR4-dependent inflammation and injury. We noted an obligatory role for integrin β3 in WISP1-mediated release of TNF-α in PM [22] and an important role for integrin β3 in polymicrobial sepsis and combined injury from poly I:C instillation and mechanical ventilation [42]. In the current study we noted that siRNA to integrin β5 reduced WISP1-mediated release of multiple cytokines in PM and integrin β5 contributed to lung inflammation and injury with CLP and MTV. Identifying the precise integrin β subunit involved in complex lung injury and signaling in isolated macrophages is complicated by: the multitude of β subunits; the nondiscriminatory inhibition by RGD peptides; and the promiscuity of WISP1 regarding interactions with αVβ integrin subunit receptors [45]. We focused on the interaction of WISP1 and integrin β5 because: others noted that WISP1 induces IL-6 production through integrin β5 receptor in human synovial fibroblasts [46]; and although several integrins are important in ALI [25], integrin β5 is a central regulator of increased permeability in VILI [26] and CLP [27].

We limited our phenotyping of lung injury to inflammation, alveolar-capillary permeability and histopathologic changes, and did not assess lung mechanics or evolution of changes in gas exchange. As such, fundamental issues of alveolar overdistension and physiologic consequences in the current study remain conjectural. It is noteworthy, however, that we recently reported [28] that low VT (6 ml/kg; 6 h) mechanical ventilation was protective after CLP (6 h). We relied on neutralizing antibodies to assess the role of WISP1 and integrin β5 in a two-hit model and partial effects noted in the study may have been secondary to incomplete deletion. For pragmatic reasons, we used PM as a surrogate for the likely cell of interest (alveolar macrophage) and future studies validating these observations with alveolar macrophages, a more challenging cell to isolate in sufficient number, are necessary.

## Conclusion

In the current study, we provide evidence that mild lung injury secondary to extrapulmonary sepsis can sensitize intact mouse lung to subsequent prolonged MTV. This two-hit model is TLR4 sensitive and has important proinflammatory contributions from both WISP1 and integrin β5. In isolated PM, we further defined the nature of WISP1 signaling and identified a requisite TLR4-dependent activation of integrin α_V_β5 and MyD88–NF-κB pathway inflammatory mediator biosynthesis. This two-hit model provides relevant new information regarding unresolved issues of a common risk factor for ARDS (systemic sepsis) and sensitization to often-used MTV.

## Additional files

Additional file 1: **Figure S1.** Flow chart of two-hit animal model: CLP followed by MTV. Mice treated as Sham group (sham CLP and sham MTV), MTV group (sham CLP followed with 6-h MTV at 10 ml/kg), CLP group (12-h CLP followed with spontaneous breathing-sham MTV) and two-hit group (12-h CLP followed with 6-h MTV). Two-hit model established by mild sepsis induced by cecal ligation and puncture (CLP) with a 22-gauge needle for 12 h followed by mechanical ventilation with moderate tidal volume at 10 ml/kg (MTV; 50% O_2_) and 150 breaths/min for 2–6 h. Two-hit model in wildtype mice compared to subgroup of TLR4 null mice (TLR4^−/−^) or wildtype mice that received intratracheally neutralizing antibodies to either integrin β5 (β5 Ab) or WISP1 (WISP1 Ab) or a control antibody (IgG Ab) during mechanical ventilation (TIF 3849 kb)

Additional file 2: **Figure S2.** Schematic of experimental groups in peritoneal macrophages. Peritoneal macrophages (PM) obtained from wildtype, TLR^−/−^ mice and treated with LPS (0.1 μg/ml) and/or siRNA to integrin β5 followed by WISP1 (10 μg/ml) exposure at 4 h. Supernatants collected at 2-h intervals from 4 to 10 h (TIF 2044 kb)

Additional file 3: **Figure S3.** MTV increases inflammatory signaling in lungs of mice after CLP. Western blot for activated (phosphorylated) p-JNK (**A**), p-p38 (**B**) and p-Erk (**C**) MAP kinase expression in lung homogenates. Mice receiving the combination of CLP + MTV (two-hit model) were compared to mice subjected to CLP alone for 18 h or sham operation followed by 6 h of MTV. Six hours of MTV alone had no effect on MAP kinase activation but significantly promoted MAP kinase activation in mice previously subjected to CLP, whereas TLR4 deletion prevented increases in MAPK activation in CLP-treated and CLP + MTV-treated mice and blocking WISP1 or integrin β5 also prevented increase in MAP kinase phosphorylation induced by MTV in CLP mice. *P < 0.05; **P < 0.01 (TIF 7656 kb)

Additional file 4: **Figure S4.** LPS-induced increase in integrin β5 expression is time and concentration dependent. Integrin β5 protein levels (**A, C**) and immunofluorescence for integrin β5 (green) and nuclei with DAPI (blue) (**B, D**) in peritoneal macrophages (PM). Mouse PM isolated from C57BL/6 mice and stimulated with LPS (0, 0.01, 0.1, 1, 10 μg/ml) in DMEM containing 10% FBS for 0–8 h. LPS induced time and concentration-dependent increase in integrin β5 protein levels and increase in surface integrin β5 expression maximal at 6 h. Corresponding actin identified for normalizing densitometry of integrin β5 expression. **P < 0.01 (TIF 1689 kb)

Additional file 5: **Figure S5.** Upregulation of integrin β5 requires TLR4/MyD88 signaling. Western blot for integrin β5 protein (**A, B)** and immunostaining of integrin β5 (**C**) in peritoneal macrophages (PM). Increase in integrin β5 protein levels and its surface expression was TLR4 and MyD88 dependent (**A**), but TRIF independent (**B**). Mouse PM isolated from C57BL/6 (wildtype), TLR4^−/−^, MyD88^−/−^ and TRIF ^−/−^ mice and stimulated with LPS (0.1 μg/ml) in DMEM containing 10% FBS for 0–6 h. Corresponding actin identified for normalizing densitometry. Immunofluorescence for integrin β5 stained green while nuclei stained with DAPI (blue). Images acquired using EVOSfl fluorescence microscopy. *P < 0.05; **P < 0.01; ***P < 0.001 (TIF 4019 kb)

Additional file 6: **Figure S6.** Integrin β5 upregulation by LPS is NF-κB dependent. Western blot for nuclear and cytoplasma NF-κB p65 (**A**) and immunostaining of NF-κB p65 (**C**) in peritoneal macrophages (PM) induced by LPS over time. Increase in integrin β5 protein levels induced by LPS at 4.5 h significantly decreased by inhibitor of NF-κB signaling, IKK-NBD (**B**). *P < 0.05; **P < 0.01; ***P < 0.001 (TIF 613 kb)

Additional file 7: **Figure S7.** siRNA to integrin β5 suppressed LPS-induced increases in integrin β5 levels in PM. Western blot for integrin β5 levels in PM after treating with siRNA to integrin β5 or control siRNA. Integrin β5 siRNA dose-dependently suppressed LPS-induced increases in integrin β5 levels compared to control siRNA. *P < 0.05; **P < 0.01; ***P < 0.001 (TIF 4824 kb)

Additional file 8 **Materials and Methods** Eight to 10-week-old male C57BL/6 mice purchased from Jackson Laboratory. TLR4^−/−^, MyD88^−/−^, TRIF^−/−^ mice obtained from Dr Billiar’s laboratory. All mice used were on a C57BL/6 background with appropriate backcrossing for respective knockouts. Transgenic male mice confirmed to be desired genotype via standard PCR-based techniques. Animal protocols approved by the Animal Care and Use Committee of the University of Pittsburgh and experiments performed in strict adherence to National Institutes of Health Guidelines for the Use of Laboratory Animals. Mice bred and housed in specific pathogen-free conditions with free access to food and water (DOCX 21 kb)

## Abbreviations

ALI: Acute lung injury
ARDS: Acute respiratory distress syndrome
CLP: Cecal ligation and puncture
EBA: Evans blue albumin
LPS: Lipopolysaccharide
MTV: Moderate tidal ventilation
MyD88: Myeloid differentiation factor 88
PM: Peritoneal macrophages
PMN: Polymorphonuclear leukocyte
RGD peptide: Arg–Gly–Asp peptide
TLR4: Toll-like receptor 4
TRIF: TIR-domain-containing adaptor inducing interferon beta
VILI: Ventilator-induced lung injury
WISP1: WNT1 inducible secreted protein
