# Supplementary figures and images for: Mechanical ventilation enhances extrapulmonary sepsis-induced lung injury: role of WISP1–αvβ5 integrin pathway in TLR4-mediated inflammation and injury
Source: Crit Care. 2018 Nov 16;22:302. doi: 10.1186/s13054-018-2237-0 (PMC6240278; doi:10.1186/s13054-018-2237-0)

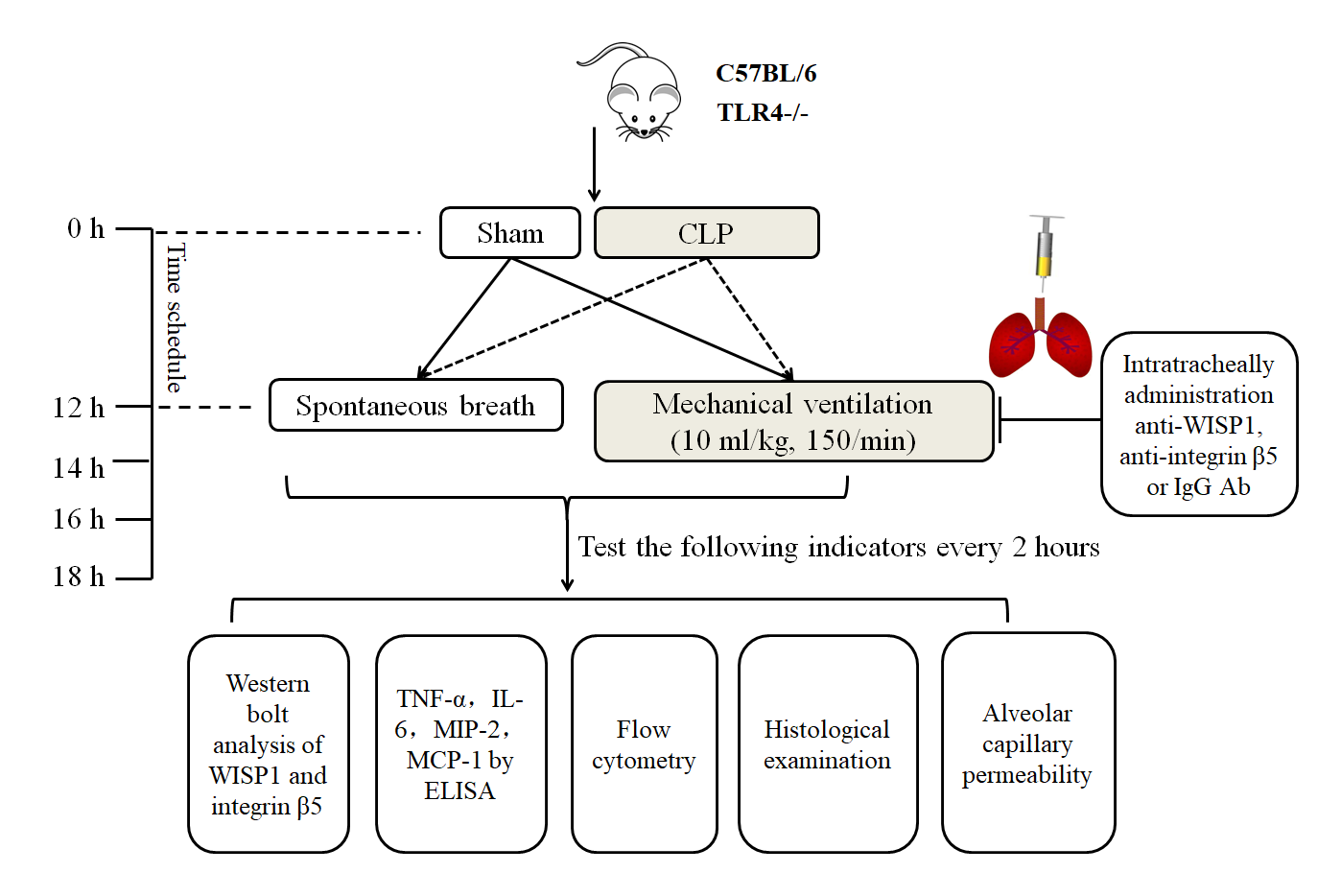

Supplement: Supplementary file 1 — Figure S1. Flow chart of two-hit animal model: CLP followed by MTV. Mice treated as Sham group (sham CLP and sham MTV), MTV group (sham CLP followed with 6-h MTV at 10 ml/kg), CLP group (12-h CLP followed with spontaneous breathing-sham MTV) and two-hit group (12-h CLP followed with 6-h MTV). Two-hit model established by mild sepsis induced by cecal ligation and puncture (CLP) with a 22-gauge needle for 12 h followed by mechanical ventilation with moderate tidal volume at 10 ml/kg (MTV; 50% O2) and 150 breaths/min for 2–6 h. Two-hit model in wildtype mice compared to subgroup of TLR4 null mice (TLR4−/−) or wildtype mice that received intratracheally neutralizing antibodies to either integrin β5 (β5 Ab) or WISP1 (WISP1 Ab) or a control antibody (IgG Ab) during mechanical ventilation (TIF 3849 kb) [file 13054_2018_2237_MOESM1_ESM.tif]

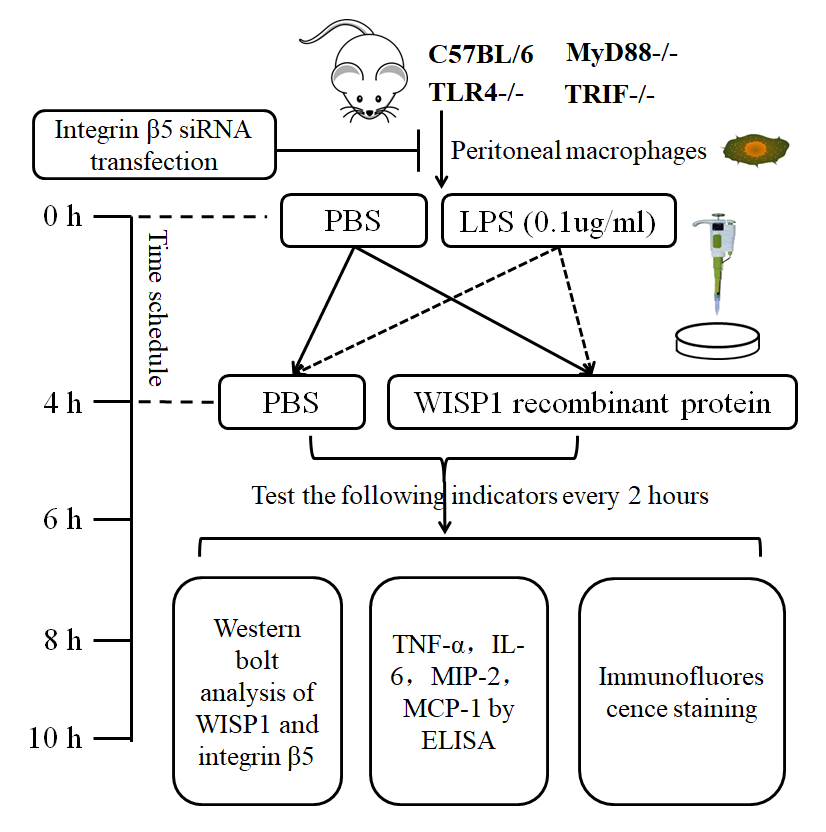

Supplement: Supplementary file 2 — Figure S2. Schematic of experimental groups in peritoneal macrophages. Peritoneal macrophages (PM) obtained from wildtype, TLR−/− mice and treated with LPS (0.1 μg/ml) and/or siRNA to integrin β5 followed by WISP1 (10 μg/ml) exposure at 4 h. Supernatants collected at 2-h intervals from 4 to 10 h (TIF 2044 kb) [file 13054_2018_2237_MOESM2_ESM.tif]

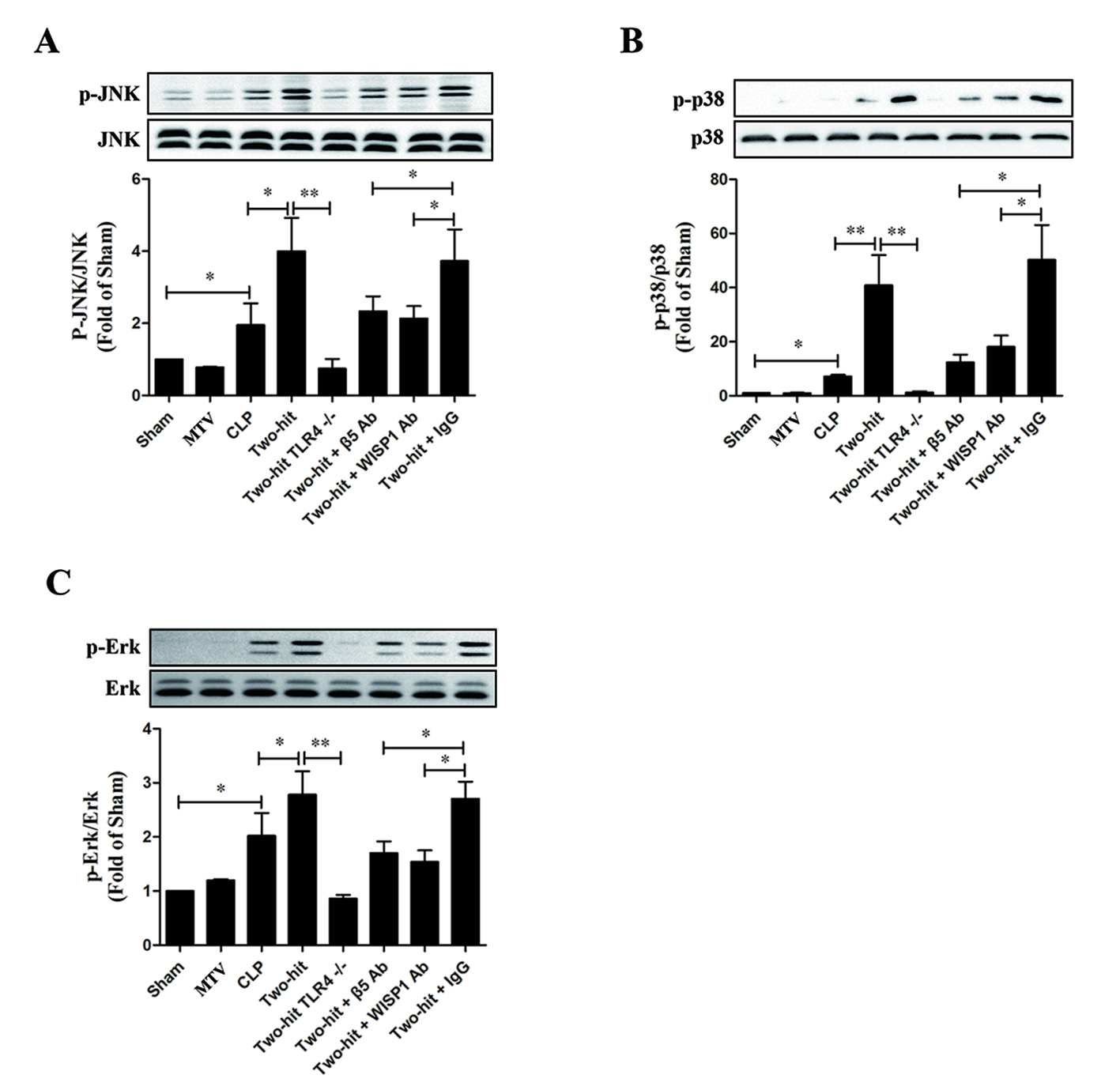

Supplement: Supplementary file 3 — Figure S3. MTV increases inflammatory signaling in lungs of mice after CLP. Western blot for activated (phosphorylated) p-JNK (A), p-p38 (B) and p-Erk (C) MAP kinase expression in lung homogenates. Mice receiving the combination of CLP + MTV (two-hit model) were compared to mice subjected to CLP alone for 18 h or sham operation followed by 6 h of MTV. Six hours of MTV alone had no effect on MAP kinase activation but significantly promoted MAP kinase activation in mice previously subjected to CLP, whereas TLR4 deletion prevented increases in MAPK activation in CLP-treated and CLP + MTV-treated mice and blocking WISP1 or integrin β5 also prevented increase in MAP kinase phosphorylation induced by MTV in CLP mice. *P < 0.05; **P < 0.01 (TIF 7656 kb) [file 13054_2018_2237_MOESM3_ESM.tif]

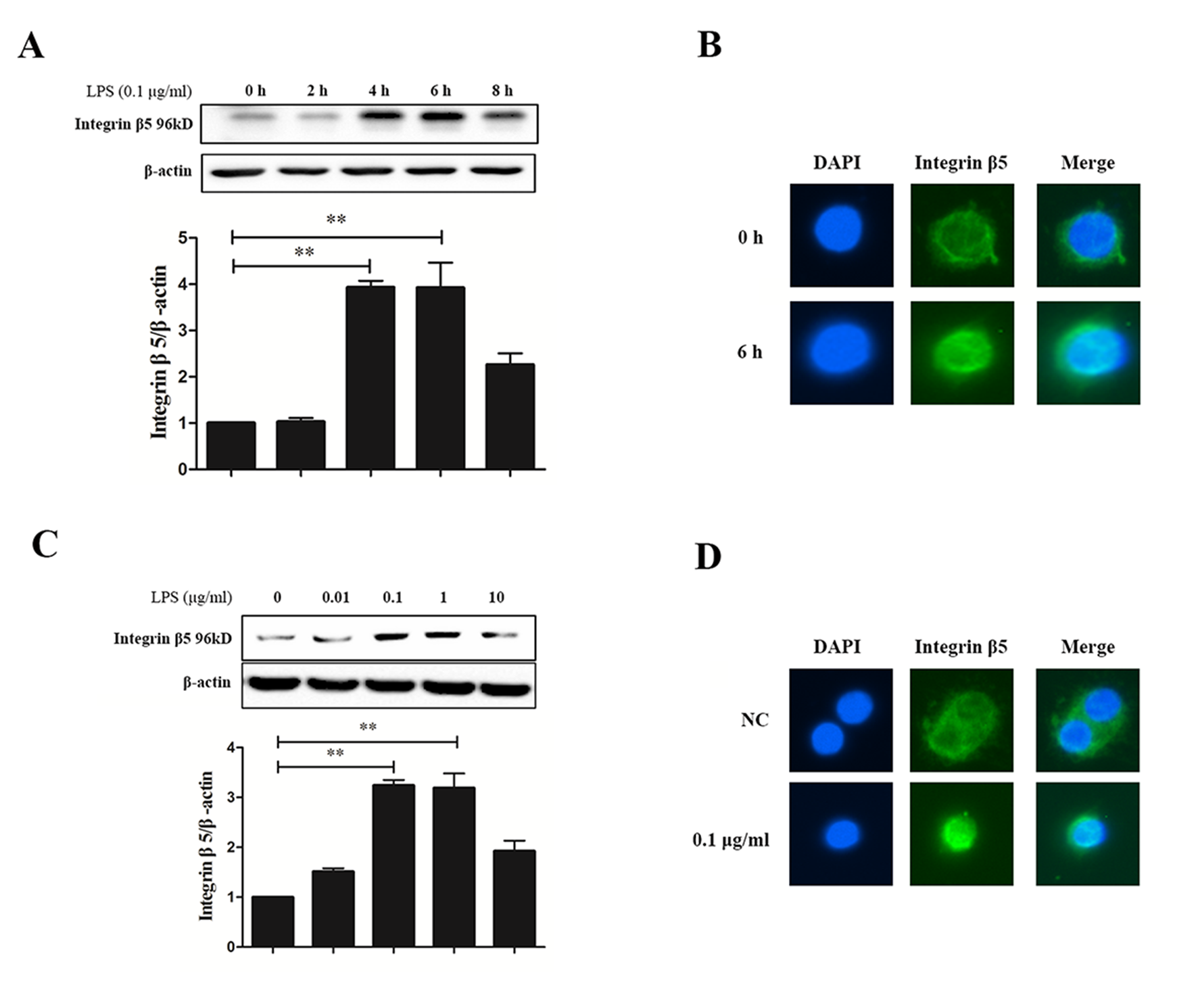

Supplement: Supplementary file 4 — Figure S4. LPS-induced increase in integrin β5 expression is time and concentration dependent. Integrin β5 protein levels (A, C) and immunofluorescence for integrin β5 (green) and nuclei with DAPI (blue) (B, D) in peritoneal macrophages (PM). Mouse PM isolated from C57BL/6 mice and stimulated with LPS (0, 0.01, 0.1, 1, 10 μg/ml) in DMEM containing 10% FBS for 0–8 h. LPS induced time and concentration-dependent increase in integrin β5 protein levels and increase in surface integrin β5 expression maximal at 6 h. Corresponding actin identified for normalizing densitometry of integrin β5 expression. **P < 0.01 (TIF 1689 kb) [file 13054_2018_2237_MOESM4_ESM.tif]

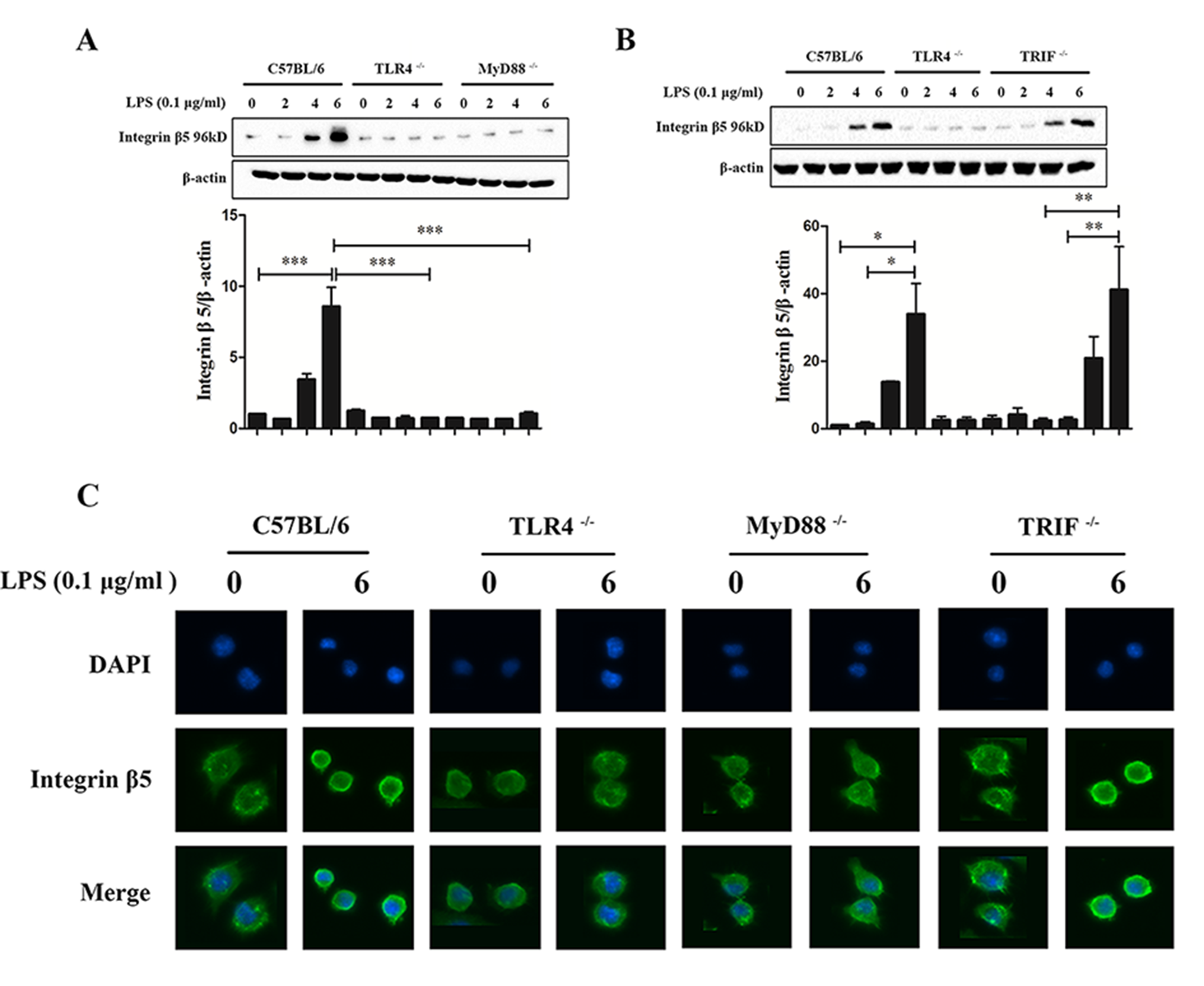

Supplement: Supplementary file 5 — Figure S5. Upregulation of integrin β5 requires TLR4/MyD88 signaling. Western blot for integrin β5 protein (A, B) and immunostaining of integrin β5 (C) in peritoneal macrophages (PM). Increase in integrin β5 protein levels and its surface expression was TLR4 and MyD88 dependent (A), but TRIF independent (B). Mouse PM isolated from C57BL/6 (wildtype), TLR4−/−, MyD88−/− and TRIF −/− mice and stimulated with LPS (0.1 μg/ml) in DMEM containing 10% FBS for 0–6 h. Corresponding actin identified for normalizing densitometry. Immunofluorescence for integrin β5 stained green while nuclei stained with DAPI (blue). Images acquired using EVOSfl fluorescence microscopy. *P < 0.05; **P < 0.01; ***P < 0.001 (TIF 4019 kb) [file 13054_2018_2237_MOESM5_ESM.tif]

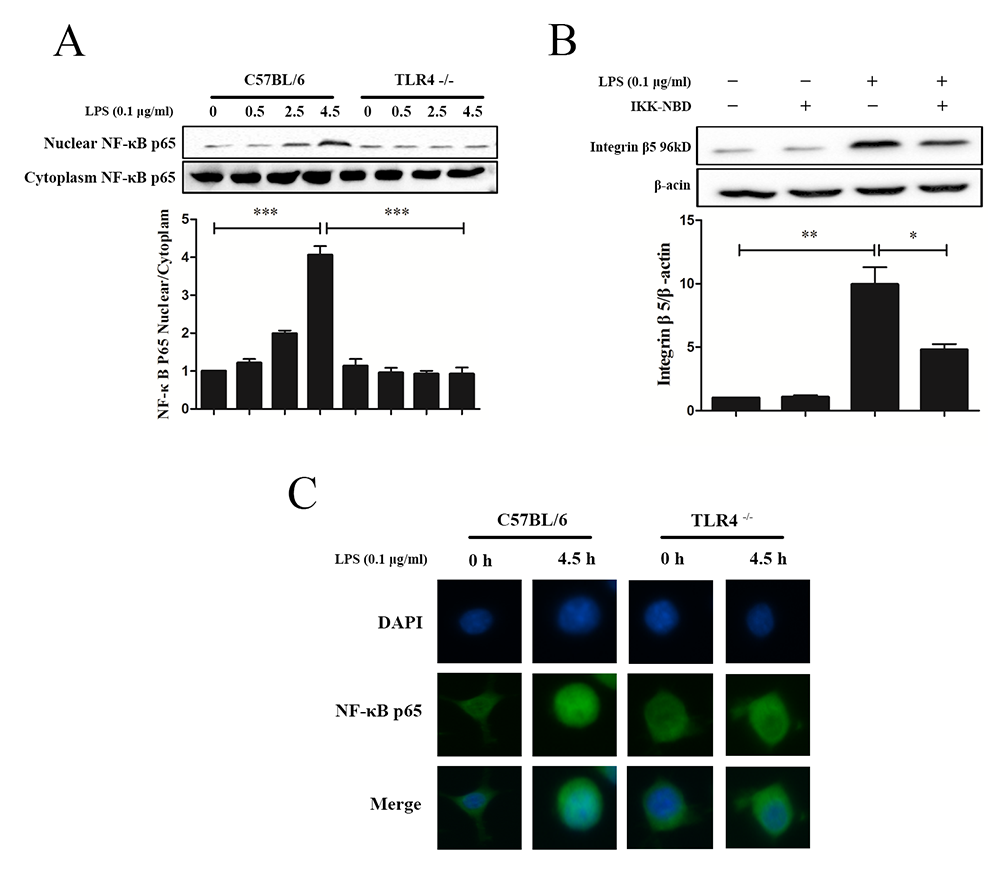

Supplement: Supplementary file 6 — Figure S6. Integrin β5 upregulation by LPS is NF-κB dependent. Western blot for nuclear and cytoplasma NF-κB p65 (A) and immunostaining of NF-κB p65 (C) in peritoneal macrophages (PM) induced by LPS over time. Increase in integrin β5 protein levels induced by LPS at 4.5 h significantly decreased by inhibitor of NF-κB signaling, IKK-NBD (B). *P < 0.05; **P < 0.01; ***P < 0.001 (TIF 613 kb) [file 13054_2018_2237_MOESM6_ESM.tif]

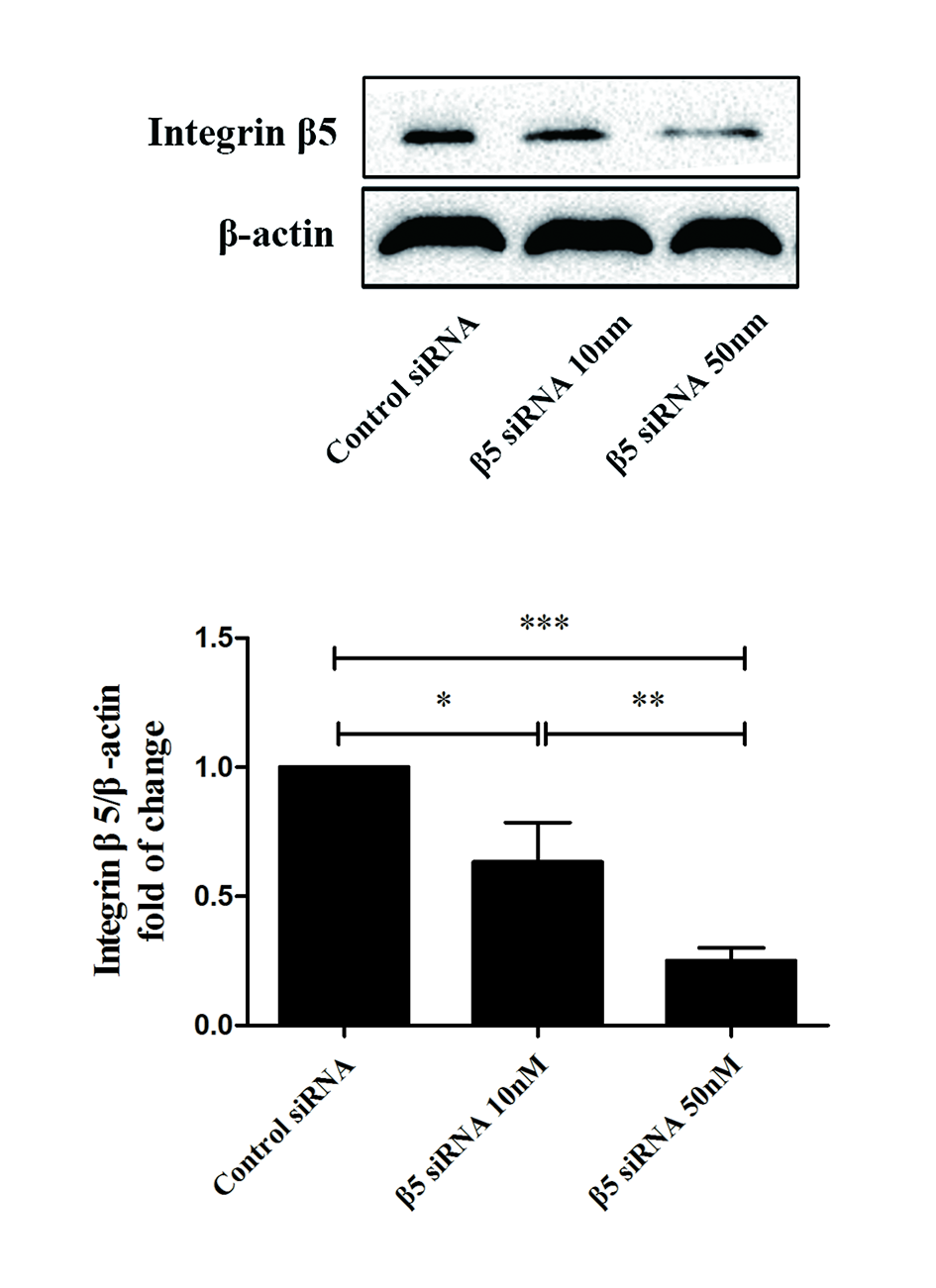

Supplement: Supplementary file 7 — Figure S7. siRNA to integrin β5 suppressed LPS-induced increases in integrin β5 levels in PM. Western blot for integrin β5 levels in PM after treating with siRNA to integrin β5 or control siRNA. Integrin β5 siRNA dose-dependently suppressed LPS-induced increases in integrin β5 levels compared to control siRNA. *P < 0.05; **P < 0.01; ***P < 0.001 (TIF 4824 kb) [file 13054_2018_2237_MOESM7_ESM.tif]
