# Supplementary material for: Mechanical ventilation enhances extrapulmonary sepsis-induced lung injury: role of WISP1–αvβ5 integrin pathway in TLR4-mediated inflammation and injury
Source: Crit Care. 2018 Nov 16;22:302. doi: 10.1186/s13054-018-2237-0 (PMC6240278; doi:10.1186/s13054-018-2237-0)
Supplement: Supplementary file 8 — Materials and Methods Eight to 10-week-old male C57BL/6 mice purchased from Jackson Laboratory. TLR4−/−, MyD88−/−, TRIF−/− mice obtained from Dr Billiar’s laboratory. All mice used were on a C57BL/6 background with appropriate backcrossing for respective knockouts. Transgenic male mice confirmed to be desired genotype via standard PCR-based techniques. Animal protocols approved by the Animal Care and Use Committee of the University of Pittsburgh and experiments performed in strict adherence to National Institutes of Health Guidelines for the Use of Laboratory Animals. Mice bred and housed in specific pathogen-free conditions with free access to food and water (DOCX 21 kb) [file 13054_2018_2237_MOESM8_ESM.docx]

**Additional file 8 – Materials and Methods**:

Mice: 8~10 week old male C57BL/6 mice were purchased from Jackson Laboratory. TLR4^-/-^, MyD88^-/-^, TRIF^-/-^ mice were obtained from Dr. Billiar’s lab. All mice used were on a C57BL/6 background with appropriate backcrossing for respective knockouts. Transgenic male mice were confirmed to be the desired genotype via standard PCR based techniques. Animal protocols were approved by the Animal Care and Use Committee of the University of Pittsburgh and experiments were performed in strict adherence to the National Institutes of Health Guidelines for the Use of Laboratory Animals. Mice were bred and housed in specific pathogen-free conditions with free access to food and water.

**Two-hit model: Mild CLP followed by MTV** Mice weighing 25~30 g were used. The mice were anesthesized by i.p. administration of 100 mg/kg ketamine and 10 mg/kg xylazine. Skin was disinfected with 2% iodine tincture. Laparotomy was performed and 50% of the cecum was ligated and punctured twice with a 22-gauge needle. The cecum was then returned to the peritoneal cavity and the abdominal incision was closed with 4-0 sterile synthetic absorbable sutures. Saline (1 ml) was given s.c. for resuscitation immediately after the operation. For analgesia, buprenorphine (0.1 mg/kg, Butler Schein, Dublin, OH) was injected to mice s.c. every 12 h staring 2 h after CLP. Mice were given antibiotics (PRIMAXIN, 25 mg/kg; Merck) s.c. every 12 h. In some experiments, anesthesized mice were subjected to MTV after CLP 12 h. An intraperitoneal injection of 1/3 initial dose of ketamine and xylazine was administered every 30 minutes to guarantee adequate anesthesia during mechanical ventilation. Body temperature was maintained at 37°C by a heating pad. Tracheotomy and intubation using 18G catheter was performed. Subsequently animals were connected to a Harvard Apparatus ventilator for 0-6 h. VT was set at 10 ml/kg and frequency was set at 150/min, zero positive end-expiratory pressure (moderate in the sense that it is slightly above range of measured VT (7 ml/kg) and respiratory rate during spontaneous ventilation). In some experiments, mice were intratracheally administered anti-WISP1, anti-integrin β5 or serum IgG (0.5 µg/g in 50 µl PBS) using a MicroSpray syringe (MicroSprayer®/Syringe Assembly for Mouse, MSA-250-M, Penn-Century, Inc.) before mechanical ventilation (Figure S1). Mice were euthanized by opening the chest cavity and withdrawal of blood by cardiac puncture.

**Isolation and culture of peritoneal macrophages (PM)**

The peritoneal cavity was injected (5-10X) with 5 ml of ice cold PBS (with 3% FBS) and the peritoneum was gently messaged to dislodge cells into the PBS solution. The suspension was aspirated and centrifuged at 1500 RPM for 8 min, and cell pellet was resuspended in Dulbecco’s Modified Eagle Medium (DMEM) supplemented with 10% fetal bovine serum (FBS), 100U/ml penicillin and 100 µg/ml streptomycin and seeded in 6/12 well plates at a concentration of 1×10^6^ cells/ml. All media were from Thermo Fisher Scientific. After overnight incubation at 37°C and 5% CO_2_, plates were washed twice with PBS to remove non-adherent cells and incubated overnight with low-serum medium (1% FBS). Cells were treated with re-purified LPS and/or co-stimulated with WISP1 recombinant protein.

**Flow cytometry**

Lung was enzymatically digested and mechanically dissociated (MACS dissociator) and single cell suspensions were isolated by passing suspension through 70-μm filter. Cells were stained with mAbs specific to Fixable Viability Dye eFluor® 506, CD45, CD11b, Ly6G for 30 minutes at 4°C and fixed with 2% paraformaldehyde for 10 minutes at 4℃. An LSR II (Becton Dickinson) was used for flow cytometry and data were analyzed with FlowJo software.

**Western blot analysis**

PM and lung tissues were lysed in buffer (Cell signaling Technology) and phenylmethylsulfonyl fluoride (PMSF). Protein concentrations were subsequently determined by standard BCA assay. After addition of 6× sodium dodecyl sulfate (SDS) loading buffer, equivalent amounts of protein were heated (100°C, 5 min) and separated by gel electrophoresis using a 10% SDS-polyacrylamide electrophoresis gel. Resolved proteins were then transferred to a nitrocellulose membrane and blocked with Tris-buffered saline containing Tween-20 (TBST) and 5% nonfat milk (1 h, 24°C). Nitrocellulose membranes were incubated overnight at 4°C with primary antibody. The membranes were washed in TBST three times, incubated with horseradish peroxidase-conjugated secondary antibody for 1 h at 37°C and then washed in TBST three additional times, before being developed for chemiluminescence (Thermo Fisher Scientific). Western blots were quantitated using Quantity One software (Bio-Rad, Foster City, CA, USA) and normalized to β-actin signal.

**Histological analyses**

Lung tissue samples were fixed in 4% paraformaldehyde in PBS overnight at 4°C. The samples were then dehydrated, embedded in paraffin, and cut into 5 μm sections. After deparaffinization, the tissues were stained with hematoxylin and eosin (H&E) for histological analysis. Lung sections were scored for lung injury, including the following: (1) alveolar and capillary edema, (2) intravascular and peri-bronchial influx of inflammatory cells, (3) thickness of the alveolar wall, and (4) hemorrhage. The items were semi-quantitatively scored as none, minimal, light, moderate, or severe (score 0, 1, 2, 3 or 4, respectively) by a pathologist blinded to the experimental group. The lung injury score was obtained by averaging the score from the animals within each group.

**Alveolar-capillary permeability**

Evans blue albumin (EBA, 0.5%, 25 mg/kg body weight) was injected into the internal jugular vein 1 h before euthanasia and lung harvesting. Blood samples were obtained from the right heart, and the pulmonary vasculature was subsequently infused with 1 mL PBS. The lung tissue was homogenized in 2 ml PBS and incubated with an additional 2 ml of formamide (Sigma-Aldrich) (18 h; 60°C). Formamide extracts were centrifuged (15,000 g × 30 min; 4°C), and the supernatants were collected to quantify lung EBA content using a dual-wavelength (620 nm and 740 nm) spectrophotometric method. Pulmonary EBA absorbance at 620 nm was corrected by a factor with EBA absorbance at 740 nm. The EBA permeability index was calculated by dividing pulmonary EBA absorbance at 620 nm/g of lung tissue by plasma EBA absorbance at 620 nm.

**ELISA**

Cell supernatants and plasma were assayed for cytokines and chemokines using commercially available ELISA reagents for TNFα, IL-6, MIP-2 and MCP-1 (Duoset, R&D System).

**Immunofluorescence staining of cells and florescence microscopy**

PM were cultured for a defined time period, fixed in 4% paraformaldehyde in PBS for 15 min. Cells were washed three timers with PBS and permeabilized using 0.1% Triton X-100 in PBS, and blocked with 5% BSA for 45 min and sequentially administered primary antibody and secondary antibody (Alexa-488-conjugated donkey anti rabbit secondary antibody). Nuclei were stained with DAPI (Thermo Fisher scientific) for immunofluoresence analysis. Stained cells were examined and recorded using EVOS FL fluorescence microscopy (Thermo Fisher scientific).

**Cell transfections**

The medium of peritoneal macrophages was replaced with serum-free, antibiotic-free DMEM prior to transfection. GeneJammer (Agilent Technologies) served as transfection reagent, and cells were transfected with 50nM small interfering RNA (siRNA) for integrin β5 (sc-35681, Santa Cruz). An unrelated, scramble small interfering RNA served as a control. Transfection efficiency was assessed 48 h posttransfection.

**Reagents:**

Ultrapure LPS (*Escherichia coli* 0111:B4) was from List Biological Laboratories (Vandell Way, CA). This LPS is free of contaminating proteins that could stimulate TLR2 nonspecifically. Anti-integrinβ5 (ab15459), anti-beta actin (ab6276), anti-WISP1 (ab178547) were from Abcam (Cambridge, MA 02139, USA); anti-phospho-SAPK/JNK (Thr183/Tyr185) (81E11), anti-SAPK/JNK, anti-phospho-p38 MAPK (Thr180/Tyr182) (D3F9), anti-p38 MAPK (D13E1), anti-phospho-p44/42 MAPK (Erk1/2) (Thr202/Tyr204) (20G11), anti-p44/p42 MAPK (Erk1/2) (137F5) were from Cell Signaling Technology (Danvers, MA 01923, USA); anti-integrinβ5 (E-19): sc-5401, anti-WISP1 (V-19): sc-8866 were from Santa Cruz Biotechnology (Santa Cruz, CA 95060, USA); mouse lung dissociation kit was from Miltenyi Biotec; Fixable Viability Dye eFluor® 506; anti-mouse CD45 PerCP-Cy5.5; anti-mouse CD11b FITC; anti-mouse Ly6G (Gr-1) APC were from eBioscience; Evans blue was from Sigma-Akdrich.
